# The ubiquitin E3 ligase activity of BRCA1 and its biological functions

**DOI:** 10.1186/1747-1028-3-1

**Published:** 2008-01-07

**Authors:** Wenwen Wu, Ayaka Koike, Takashi Takeshita, Tomohiko Ohta

**Affiliations:** 1 Division of Breast and Endocrine Surgery, St. Marianna University School of Medicine, Kawasaki, 216-8511, Japan

## Abstract

The basal-like breast cancer, a new category of breast cancer associated with poor prognosis and possibly unique chemosensitivity, is a current topic in the breast cancer field. Evidence from multiple sources strongly indicate that impairment of BRCA1 pathways is responsible for this phenotype, implying the importance of BRCA1 not only in familial breast cancers but also in sporadic cancers. BRCA1 acts as a hub protein that coordinates a diverse range of cellular pathways to maintain genomic stability. BRCA1 participates in multiple cellular supercomplexes to execute its tasks and, in most of the complexes, BRCA1 exists as a RING heterodimer with BARD1 to provide ubiquitin E3 ligase activity that is required for its tumor suppressor function. It was revealed recently that the BRCA1 RING finger is capable of catalyzing multiple types of ubiquitination depending upon the interacting E2, the ubiquitin carrier protein. BRCA1 may catalyze distinct ubiquitination on different substrates as the situation demands. On the other hand, in response to DNA double-strand breaks where BRCA1 plays its major role for homologous recombination repair, recent evidence showed that ubiquitination is a critical step to recruit BRCA1 to the damaged site through UIM (ubiquitin interacting motif) containing protein RAP80. Thus, ubiquitin and BRCA1 likely affect each other in many ways to perform cellular functions. Elucidation of this mechanism in relation to cell survival is now much anticipated because it could be a key to predict chemosensitivity of basal-like breast cancer.

## Introduction

The breast and ovarian cancer susceptibility gene *BRCA1 *is implicated not only in familial breast cancers but also in sporadic breast cancers. Recently, the role of BRCA1 in sporadic cancers became more apparent. Gene expression profiling studies by high-throughput microarray analyses have succeeded in categorizing breast cancer into several subsets that are associated with distinct clinical outcomes, including luminal A, luminal B, normal breast-like, HER2 overexpressing, and basal-like [1,2]. Approximately 15% of sporadic breast cancers are categorized into the basal-like subtype that expresses basal/myoepithelial cell markers, but does not express estrogen receptor (ER), progesterone receptor (PR), or HER2 [1-3], and is associated with poor prognosis. Impairment of BRCA1 pathways is likely responsible for this subtype. For example, a majority of hereditary breast cancers with BRCA1 mutations display a basal-like phenotype [2,4-7], BRCA1 dysfunction in sporadic basal-like cancers has been reported [8-10], and conditional deletion of BRCA1 and p53 in the mammary gland of mice results in a phenotype reminiscent of human basal-like breast cancer [11,12]. Because BRCA1's function is implicated in the chemosensitivity of cells to DNA damage-inducing reagents [13,14], elucidating the mechanisms of the BRCA1 pathways is now a critical issue directly linked to the clinical breast cancer field.

When considering BRCA1's functions it should be remembered that it is an enzyme, a ubiquitin E3 ligase [15-17]. In addition, it was reported recently that polyubiquitin chains recruit BRCA1 to damaged DNA sites [18-21], suggesting that ubiquitination plays key roles both upstream and downstream of the BRCA1 pathways. However, the role of BRCA1's E3 activity in executing its cellular functions is still unclear. This review summarizes BRCA1's function in the context of its relationship to ubiquitin and discusses the possible roles of how the activity is involved in its biological functions.

### BRCA1 in multiple cellular supercomplexes

**Figure 1:**
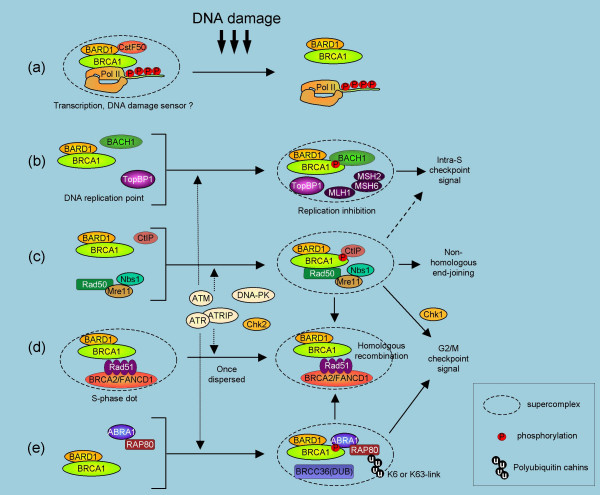
**BRCA1 supercomplex formation in response to DNA damage**. BRCA1 acts in many cellular supercomplexes and executes distinct roles. BARD1 stoichiometrically interacts with BRCA1 and exists as a RING heterodimer in all the complexes shown, indicating the importance of E3 ligase activity in each complex. (a) BRCA1-BARD1 in the Pol II-holoenzyme may act as a transcriptional co-activator. In addition, BRCA1 that interacts with hyperphosphorylated Pol II acts as a sensor for DNA damage. After some types of DNA damage, BRCA1 dissociates with Pol II. BRCA1-BARD1 may polyubiquitinate subunits of this complex at this time, including the hyperphosphorylated largest subunit (RPB1) and the common small subunit (RPB8). (b) BRCA1 that interacts with BACH1/BRIP1/FANCJ at its C-terminus locates the replication point with BARD1. This complex interacts with TopBP1 in an ATM dependent manner after DNA damage and causes TopBP1 dissociation from the replication point. This results in the failure to recruit the replication initiation factor Cdc45, likely engaging the intra S-phase checkpoint. (c) Mre11-Rad50-Nbs1 also forms a complex with BRCA1-BARD1 at sites of DNA damage. This BRCA1 complex interacts with CtIP via the BRCA1 C-terminus. Complex formation depends on ATM- and Chk2-mediated phosphorylation. This complex signals the G2/M checkpoint and bridges the two DNA ends of the DSB, an intermediate of both non-homologous end joining (NHEJ) and homologous recombination (HR). (d) BRCA1 constitutes S-phase nuclear foci with BARD1, Rad51 and BRCA2. After DNA damage, these once disperse foci again localize to damaged DNA sites. BRCA1 is necessary for recruiting these proteins. This complex is important for homologous recombination repair of DSB. (e) BRCA1-BARD1 is targeted to polyubiquitin chains at DNA damaged sites through BRCT domain-interacting phosphorylated ABRA1 that interacts with UIM containing protein RAP80.

BRCA1 acts as a hub protein that coordinates a diverse range of cellular pathways including DNA repair, cell cycle control, transcriptional regulation, apoptosis, and centrosome duplication to maintain genomic stability. BRCA1 participates in several different cellular supercomplexes to coordinate these functions, and each complex contains only a small fraction of the total cellular BRCA1 [22]. For example BRCA2 interacts with only 5% or less of the total BRCA1 [23]. Representative complexes include RNA polymerase II holoenzyme complex (Pol II) [24], Mre11-Rad50-Nbs1 (MRN) complex [25], BRCA2-Rad51 complex [23,26], BACH1 (also called BRIP1 or FANCJ)-TopBP1 complex [22,27], SWI/SNF chromatin remodeling complex [28], and a complex with γ-tubulin in the centrosome [29]. Representative BRCA1 supercomplexes that respond to DNA damage are shown in Figure 1.

**Figure 2:**
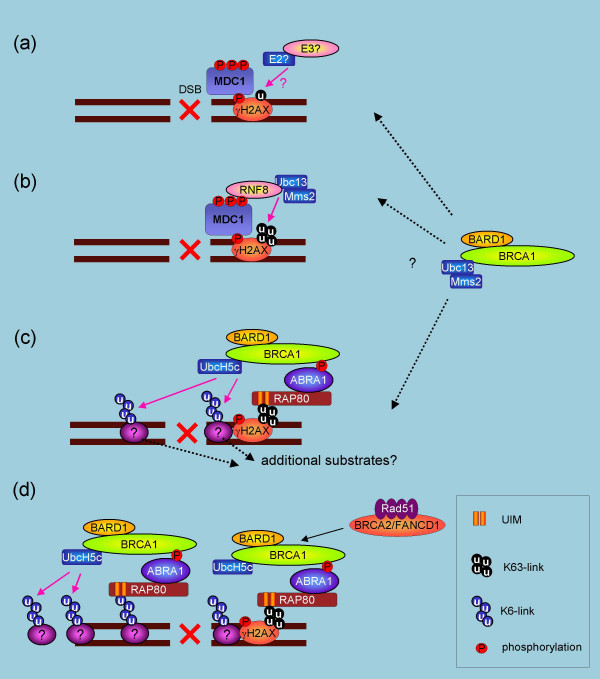
**Models for ubiquitin accumulation at DSB**. Upon DSBs, γH2AX could be monoubiquitinated (a) so that RNF8-Ubc13-Mms2 can attach additional Lys63-linked polyubiquitin chains on the ubiquitin (b). The E2 and E3 for monoubiquitination are unknown. BRCA1-BARD1 is then targeted to the polyubiquitin chains through RAP80-ABRA1 in a manner dependent on ABRA1 phosphorylation. BRCA1-BARD1 interacts with UbcH5c and mediates further polyubiquitin chains, including Lys6-linked chains (c). The BRCA1-BARD1-induced polyubiquitin chains further recruit additional BRCA1-BARD1 complexes to damaged sites, resulting in focal accumulation of polyubiquitin chains (d). The polyubiquitin chain assembly is likely required for BRCA2-Rad51 recruitment to damaged sites, allowing execution of homologous recombination repair. Recent studies suggest that the BRCA1-BARD1 heterodimer could also catalyze substrate monoubiquitination with substrate-specific E2s, such as UbcH6, Ube2e2, UbcM2, and Ube2w, as well as additional Lys63-linked polyubiquitin chains with Ubc13-Mms2. The substrate(s) and the timing of this action remain to be determined.

BRCA1 interacts with many transcriptional cofactors and regulates transcription. For example, BRCA1 stimulates p53-dependent transcription of p21^cip1/WAF1 ^[30-32]. BRCA1 that interacts with Pol II may act as a transcriptional coactivator of some specific genes. Alternatively, BRCA1 in the elongating Pol II complexes may also act as a sensor for DNA damage [33,34]. In this model, BRCA1 dissociates from Pol II after certain types of DNA damage, including those caused by the topoisomerase inhibitors [33] or UV irradiation (our unpublished data) (Fig 1a). On the other hand, BRCA1 associates with several different protein supercomplexes in response to DNA double-strand breaks (DSB) such as that introduced by ionizing radiation (IR) or replication stress [22]. BRCA1 comprises a complex with BRCA2 and Rad51 that mediates HR of the DSBs (Fig 2d) [35-39]. BRCA1 also interacts with MRN after DNA damage in a manner dependent on checkpoint kinases ATM or Chk2 (Fig 2c) [22,25,40]. MRN mediates the G2/M checkpoint signal and plays roles in DSB by holding two DNA ends close to each other. This process is important for non-homologous end-joining function and also precedes BRCA2-Rad51 mediated HR [14]. BRCA1 that interacts with MRN binds to CtIP through the C-terminal BRCT domain [22]. The BRCT domain of BRCA1 also interacts with phosphorylated BACH1, ABRA1, and ATRIP [18,22,41-43]. BRCA1-BACH1 interacts with TopBP1 in an ATM-dependent manner after DNA damage and causes TopBP1 dissociation from the replication point, resulting in a failure to recruit replication initiation factor Cdc45 [22] (Fig 2b). This complex may be implicated in intra-S phase checkpoint. ABRA1, also called coiled-coil containing protein CCDC98, mediates the interaction between BRCA1 and RAP80, which recruits BRCA1 to damaged DNA sites through its ubiquitin-interacting motif (UIM), as described below [18] (Fig 2e). Importantly, amongst its many protein binding partners only BARD1 interacts stoichiometrically with BRCA1 in cells and presumably exists as a BRCA1-BARD1 RING heterodimer in most complexes [22]. Because this dimer formation results in E3 ubiquitin ligase activity [15,17,44], BRCA1 likely acts as an enzyme in many cellular complexes and directs the ubiquitination of distinct substrates within each complex.

### BRCA1-BARD1 RING heterodimer E3 ligase

The ubiquitin system consists of three critical enzymes: a ubiquitin-activating enzyme (E1), a ubiquitin-conjugating enzyme (E2), and a ubiquitin ligase (E3). The E3 catalyzes the formation of polyubiquitin chains (or sometimes monoubiquitin), utilizing ubiquitins that have been activated by the E1 and E2 enzymes, and transfers them onto specific substrate(s) via isopeptide bonds [45,46].

The *BRCA1 *gene encodes a protein of 1,863 amino acids with a molecular weight of 220 kDa [47]. The protein contains an N-terminal RING finger domain, a common E2 binding motif found in E3 ligases, and tandem BRCT domains in its C-terminal region. The N-terminal RING finger domain of BRCA1 interacts with another conformationally similar RING finger protein, BARD1, that contains an N-terminal RING domain and C-terminal tandem BRCT domains [48] to constitute a RING heterodimer E3 ligase [15,17,44]. The BARD1 interaction is required to stabilize the proper conformation of the BRCA1 RING domain for E3 activity [44]. The RING finger of BRCA1 interacts with E2 UbcH5 whereas the role of the BARD1 RING domain is currently unknown. The BRCT repeats constitute a phosphopeptide recognition domain that binds peptides containing a phospho-SXXF motif [41,42]. Because BRCA1 BRCT domains form mutually exclusive complexes with CtIP, BACH1, and ABRA1 in a phosphorylation-dependent manner, it has been proposed that these proteins may serve as adaptor proteins to target the BRCA1-BARD1 E3 ligase to specific substrates [18,49]. In this regard, these complexes resemble the SCF (Skp1, CUL1, F-box) complex where the N-terminus of CUL1 interacts via Skp1 with F-box adaptor proteins that recruit specific substrates in a phosphorylation dependent manner while the C-terminus of CUL1 interacts with the RING subunit ROC1/Rbx1 [50-53]. Identification of substrates ubiquitinated by BRCA1-BARD1 in such a structurally similar manner is of great interest. On the other hand, the other regions of BRCA1, including that encoded by the largest coding exon 11, as well as N-terminal regions adjacent to the RING domain, also bind to many critical cellular proteins, some of which have been reported to be ubiquitinated by BRCA1-BARD1.

### Substrate recognition sites of BRCA1

Putative substrates of BRCA1-BARD1 identified at present are histones, γ-tubulin, ERα, nucleophosmin/B23 (NPM1), the largest subunit of RNA polymerase II (RPB1), CtIP, the common subunit of RNA polymerases (RPB8), progesterone receptor-A (PR-A), and the general transcription factor TFIIE [49,54-62].

The substrate recognition sites for the majority of these substrates are distributed throughout the N-terminus half of BRCA1. Ubiquitination of histone H2A and H2AX requires N-terminal residues 1–639 of BRCA1 [54]. The hyperphosphorylated forms of full-length RPB1 (RNAPIIO) can be polyubiquitinated by N-terminal residues 1–304 of BRCA1 in vitro[55]. However, it has also been reported that the C-terminus of BRCA1 is required for efficient RPB1 ubiquitination in vivo despite the result that N-terminal residues 1–500 of BRCA1 are sufficient to ubiquitinate the C-terminal domain (CTD) of RPB1 in vitro [56]. NPM1 and RPB8 were also ubiquitinated by the N-terminal constructs of BRCA1 in vivo and in vitro [57,58]. Although NPM1 was only poorly ubiquitinated with recombinant BRCA1 residues 1–304 and BARD1 residues 14–189 [57], it was effectively polyubiquitinated with residues 1–772 of BRCA1 and full-length BARD1 in vitro (unpublished data), suggesting that the BRCT domains of BRCA1 are dispensable for this substrate. In some cases substrate recognition appears to occur via a combination of BRCA1 and BARD1 sequences. For example, ubiquitination of ERα requires N-terminal BRCA1 residues 1–241 in combination with N-terminal BARD1 residues 1–170 [59]. In contrast substrates recognized by the C-terminus of BRCA1 include γ-tubulin and CtIP. Full-length BRCA1, including C-terminal residues 1852–1863, was necessary for γ-tubulin monoubiquitination, although the domains required for γ-tubulin binding reside in the amino-terminal 803 amino acids of BRCA1 [60]. CtIP interacts with BRCT and is polyubiquitinated by BRCA1-BARD1 in a phosphorylation dependent manner. In this case, the BRCT domains play the role of a substrate recruitment adaptor, the function similar to the F-box proteins within the SCF complex [49]. From this perspective, it could be interesting to test whether the BRCT binding proteins BACH1 and ABRA1 are also ubiquitinated by BRCA1.

### Types of ubiquitination and signals

Biological consequences for the ubiquitination of the above substrates are not yet completely understood. One of the reasons for this difficulty is that the signal mediated by BRCA1-induced ubiquitination has not been determined. Depending upon the type of ubiquitin chain, ubiquitin modifications signal a variety of processes [63-65]. The most common polyubiquitin chains are linked through Lys48 of ubiquitin and serve as a signal for proteolysis by the proteasome [66]. However, BRCA1-BARD1 catalyzes unconventional polyubiquitin chains that include Lys6-linked chains [67-69]. Putative degradation substrates reported at present are RNAPIIO and PR-A [55,61]. Because double knock-down of BRCA1 and BARD1 restored the expression level of the RNAPIIO that had been repressed by UV irradiation, it was proposed that BRCA1-BARD1 could initiate the degradation of stalled RNAPIIO [55]. However, the BRCA1/BARD1 double knock-down did not detectably affect RPB1 ubiquitination after UV irradiation. Therefore, the restored expression level of RNAPIIO by BRCA1/BARD1 double knock-down could be due to an indirect effect. PR-A is stabilized in the absence of BRCA1 in knockout mice as well as in siRNA knock-down cells [61]. Importantly, anti-progesterone treatment dramatically inhibits breast carcinogenesis in mice defective for BRCA1 and p53, indicating that downregulation of PR-A by BRCA1 is critical to prevent breast cancer. In this process, PR-A has been shown to be ubiquitinated by BRCA1 overexpression in vivo. However, the BRCA1/BARD1 complex did not ubiquitinate PR-A in vitro [61]. Therefore, to date there is no direct evidence supporting the notion that BRCA1-mediated ubiquitination signals degradation.

On the other hand, numerous substrates have been shown not to be degraded by BRCA1-catalyzed polyubiquitination: NPM1, CtIP, RPB8, TFIIE and BRCA1 itself [49,57,58,62,67]. BRCA1 autoubiquitinates when bound to BARD1, and the ubiquitination stabilizes BRCA1 and enhances its E3 activity, suggesting a positive feedback effect for activity [15,67,70]. Although detailed biochemical mechanisms regarding the ubiquitination signal remain to be elucidated, the ubiquitination of these substrates results in some biological effects. Ubiquitinated CtIP associates with chromatin after γ-radiation, and this ubiquitination could be important for CtIP focus formation at the DNA damaged site. This is because only wild-type BRCA1, but not the I26A E3 ligase mutant of BRCA1, restored DNA damage-induced CtIP focus formation in BRCA1-deficient HCC1937 cells [49]. Consistent with a role for the BRCA1-CtIP complex in G2/M checkpoint control, BRCA1-I26A did not restore the G2/M checkpoint of HCC1937 cells while wild type BRCA1 did. In contrast to the chromatin association of ubiquitinated CtIP, BRCA1 ubiquitination of TFIIE, a subunit of the basal transcription factor, causes its dissociation from the transcription pre-initiation complex at the promoter and blocks the initiation of mRNA synthesis [62]. In addition to the basal transcription factor, the common subunit of all three RNA polymerases, RPB8, is also polyubiquitinated by BRCA1 [58]. RPB8 was polyubiquitinated immediately after ultra-violet (UV) irradiation, and this ubiquitination was inhibited by BRCA1 knock-down via RNA interference. BRCA1 knock-down also resulted in decreased RPB8 protein in the soluble cellular fraction, suggesting that BRCA1 ubiquitination of RPB8 may cause dissociation of RPB8 from chromatin-associated complexes in a fashion similar to TFIIE dissociation from the pre-initiation complex. The ubiquitination of RPB8 is likely not important for normal transcriptional processes because a ubiquitin-resistant form of RPB8 preserves its polymerase activity and HeLa cells stably expressing this mutant grow normally. However, this cell line exhibited UV hypersensitivity [58], suggesting that ubiquitination of RPB8 is important for BRCA1-dependent cell survival after DNA damage, which could be caused by inhibition of synthesis of mRNA or other RNAs. The biological significance of NPM1 ubiquitination by BRCA1 is currently unknown. Because NPM1 has multiple cellular functions implicated in BRCA1's function, such as histone chaperone activity, licensing of centrosome duplication, regulation of the ARF-MDM2-p53 pathway and inhibition of apoptosis, it will be important to investigate the role of BRCA1 ubiquitination of NPM1 in these biological functions.

BRCA1 also catalyzes the monoubiquitination of some substrates. Core histones H2A, H2B, H3, and H4 are all monoubiquitinated in vitro [54,70]. In addition, phosphorylated DNA damage responsive histone variant H2AX (γH2AX) is also monoubiquitinated in vitro [54,70]. Although the biological significance of this monoubiquitination is poorly understood, it could contribute to chromatin remodeling in BRCA1-dependent DNA repair pathways. γ-tubulin is also monoubiquitinated by BRCA1 in vitro and mutation of the monoubiquitination site on γ-tubulin results in centrosome hyperamplification, suggesting that BRCA1 regulates centrosome number and proper nuclear segregation through γ-tubulin monoubiquitination [60]. Another substrate reported to be monoubiquitinated is ERα [59]. The ligand-binding domain of ERα is monoubiquitinated by BRCA1-BARD1 in vitro.

### E2 specificity of the BRCA1 RING

As the above discussion makes clear, ubiquitination by BRCA1 is a diverse process, likely with different fates for different substrates. One question that arises is how BRCA1 catalyzes its different ubiquitination products. A clue to this conundrum has recently been reported, showing that specific E2-BRCA1 RING interactions determine the type of ubiquitination [71]. Surprisingly, the RING finger domain of BRCA1 is capable of interacting with UbcH6, UbcH7, Ube2e2, UbcM2, Ube2w, Ubc13, and Ube2k in addition to UbcH5, the E2 previously known to interact with BRCA1, through conserved residues located in Helix 1 and Loop L2 of each E2. Mutation of key residues in these regions, such as A96D in Loop L2 of UbcH5c, eliminates the interaction. BRCA1-BARD1 in combination with UbcH6, Ube2e2, UbcM2, or Ube2w resulted in monoubiquitination of BRCA1 while in combination with UbcH5c resulted in polyubiquitination. In contrast, incubation of BRCA1-BARD1 with UbcH7, Ubc13-Mms2 or Ube2k did not catalyze ubiquitin transfer from the E2s, even though they bind to the BRCA1 RING. Unexpectedly, incubation of BRCA1-BARD1 with Ubc13/Mms2 or Ube2k in the presence of Ube2w resulted in polyubiquitination of BRCA1 that was perturbed by K63R or K48R mutation of ubiquitin, respectively. The nature and location of the non-covalent interaction between the acceptor monoubiquitinated ubiquitin molecule on BRCA1 and either Mms2 or the C-terminus of Ubek2 play critical roles for polyubiquitination assembly and the determination of the specific chain linkages. Based on the above observations, Christensen et al. [71] classified Ubc13-Mms2 and Ube2k as ubiquitin-specific E2s for BRCA1 and classified UbcH6, Ube2e2, UbcM2 and Ube2w as substrate-specific E2s for BRCA1. The ubiquitin-specific E2s conjugate ubiquitin to another ubiquitin, while the substrate-specific E2s transfer ubiquitin directly to the BRCA1 substrate, but not to ubiquitin itself. UbcH5c is unique in its ability to both transfer the first ubiquitin to BRCA1 and extend the chain. The discovery of this E2-BRCA1 RING specificity may contribute to progress in many aspects of research into BRCA1 function. For example, BRCA1's ability to catalyze Lys48-linked chains may explain the BRCA1-mediated ubiquitination of and proteasome-dependent degradation of RNAPIIO and PR-A, as described above [55,61]. It is also possible that the previously known monoubiquitinated substrates, such as histones and γ-tubulin, could be substrates for polyubiquitination with additional E2s. For instance, γH2AX is only monoubiquitinated in vitro in previously reported reactions using a single E2 [54,70] although it can be polyubiquitinated in vivo in response to γ-radiation in a Ubc13-dependent manner [72]. In addition, many candidate substrates may have been discarded in previous *in vitro *studies that used only UbcH5 as the E2. Assay of such candidate substrates with BRCA1-BARD1 and the panel of E2s with which it works may reveal overlooked targets.

### Polyubquitin chain recruits BRCA1 to damaged DNA sites

One of the most important functions of BRCA1 is its role in DNA double-strand breaks (DSBs). BRCA1 and BARD1 are required to recruit Rad51 and BRCA2, the central players in HR at damaged DNA sites [22,35-39]. The E3 ligase activity of BRCA1 could be critical in this function. However, the collaboration between BRCA1 and ubiquitin goes beyond this relationship. It was reported recently that BRCA1 is recruited to the DSBs by polyubiquitin chains through RAP80, a ubiquitin interacting motif (UIM) containing protein that had previously been characterized as an ERα-interacting nuclear protein [18-21,73](Figure 2). RAP80 constitutes a protein complex with ABRA1 that interacts with BRCT domain of BRCA1 in a phosphorylation dependent manner [18]. Upon DSBs, RAP80-ABRA1 targets the BRCA1-BARD1 complex and the deubiquitinating (DUB) enzyme BRCC36 to Lys6- and Lys63-linked polyubiquitin chains at DSBs [18,19]. These polyubiquitin chain assemblies were MDC1- and γH2AX-dependent [19]. Strikingly, the substrates of the polyubiquitin chains, as well as the E3 that catalyzed them, were recently determined to be H2AX and RNF8, respectively [74-77]. RNF8 contains an N-terminal forkhead-associated (FHA) domain and a C-terminal RING domain. While RNF8 is recruited to MDC1 through a phosphorylation-dependent interaction between the FHA domain and motifs in MDC1 that are phosphorylated by ATM, the RING domain polyubiquitinates H2AX or H2A. Knockdown of RNF8 by siRNA impaired IR-induced polyubiquitination of H2AX [75] and inhibited BRCA1's focus formation at DSB sites [74-77], indicating that RNF8 acts upstream of BRCA1. Because knockdown of Ubc13, an E2 known to form K63-linked polyubiquitin chains, also inhibited BRCA1 and RAP80 focus formation [74-77] and because RNF8 interacted with Ubc13 [78], it is likely that the E2 cooperating with RNF8 in the pathway is Ubc13. However, *in vitro *H2A or H2AX polyubiquitination mediated by RNF8 and Ubc13 remains to be shown. Only polyubiquitination of histones mediated by RNF8 and UbcH5c has been demonstrated at present [74].

It was also previously reported from another group that Ubc13-dependent polyubiquitination is required for HR [72]. A complex comprised of the E2 Ubc13-Mms2 and E3 Rad5 complex catalyzes Lys63-linked polyubiquitin chains on the Rad6/Rad18-induced monoubiquitinated PCNA that is implicated in post-replication repair in budding yeast [79-81]. In vertebrates, however, it was shown using Ubc13-deficient DT40 cells that Ubc13 is required to repair a wide range of DNA-damage including that caused by IR, UV, H_2_O_2_, and DNA crosslinking or DSB-causing chemical reagents [72]. Ubc13-deficient DT40 cells exhibited prolonged γH2AX foci formation after IR accompanied by loss of Rad51 and conjugated-ubiquitin containing foci formation (detected by FK2 antibody), suggesting that the Ubc13-mediated ubiquitination is required for Rad51-mediated HR. Importantly, siRNA knock-down of Ubc13 in HeLa cells eliminated E3 ligase activity of BRCA1 immunocomplexes precipitated from IR treated cells when incubated with ubiquitin, E1 and ATP, but not with E2. Because the immunocomplex's loss of E3 ligase activity was accompanied by dissociation of IR-induced interaction of UbcH5c with the complex, it was speculated that Ubc13 activates BRCA1's E3 activity by enhancing the BRCA1-UbcH5c interaction [72]. Therefore, one possible model is that upon DSBs, RNF8-Ubc13-Mms2 catalyzes Lys63-linked polyubiquitin chains on γH2AX (Figure 2). RAP80-ABRA1 targets BRCA1-BARD1 to the polyubiquitin chains where UbcH5c interacts with BRCA1 and catalyzes Lys6-linked polyubiquitin chains. The Lys6-linked polyubiquitination might be recognized again by RAP80 to cause auto-amplification of the ubiquitination signal at the DNA damaged focus. Polyubiquitin chain assembly is required for further processes of HR, including RPA binding to single strands and recruitment of Rad51 [72]. In this model, an E2 and an E3 that mediate monoubiquitination of γH2AX prior to RNF8-Ubc13-Mms2-mediated Lys63-linked polyubiquitination could be required. An additional candidate E3 that interacts with Ubc13 in the pathway includes the human homologue of Rad5, SHPRH, that was recently identified to interact with Rad6-Rad18 and Mms2-Ubc13 and to polyubiquitinate PCNA [82]. However, PCNA likely is not the Lys63-linked polyubiquitinated substrate of Ubc13-dependent ubiquitination in DSBs. This is because Rad51 focus formation after IR was not perturbed in the DT40 cells in which Lys165 of PCNA (the Rad6-Rad18- and Mms2-Ubc13-dependent ubiquitination site) was replaced by arginine [72]. Other E3s that interact with Ubc13 include CHFR and TRAF6 [83,84]. Furthermore, now it is known that Ubc13 can interact directly with BRCA1 RING and is capable of assembling Lys63-linked polyubiquitin chains on monoubiquitinated substrate [71]. How and when BRCA1-BARD1 cooperates with a substrate-specific E2, such as UbcH6, Ube2e2, UbcM2, or Ube2w, as well as a ubiquitin-specific E2, such as Ubc13/Mms2 or Ube2k, would be an interesting matter to be determined. In this regard, deficiencies in some of these E2s may also affect HR after DSBs.

## Conclusion

BRCA1-BARD1 likely acts as an E3 in many cellular complexes and directs the ubiquitination of distinct substrates within each complex. Its putative substrates identified at present are histones, γ-tubulin, ERα, NPM1, RPB1, RPB8, CtIP, PR-A, and TFIIE. Ubiquitination by BRCA1 is a diverse process, likely with different fates for different substrates. Incubation of BRCA1-BARD1 with ubiquitin-specific E2s, such as Ubc13/Mms2 or Ube2k, in the presence of substrate-specific E2s, such as Ube2w, resulted in K63-linked or K48-linked polyubiquitin chains, respectively, in addition to K6-linked polyubiquitin chains mediated by UbcH5. These *in vitro *results remain to be fully validated. On the other hand, the ubiquitination-dependent BRCA1 recruiting mechanism after DSB has been elucidated, and γH2AX polyubiquitination, RNF8, Ubc13, RAP80, and ABRA1 were involved in the system. Turnover of polyubiquitinated structures by DUB activity, such as by BRCC36 or BAP1, may further regulate this reaction. Thus, many actors have appeared on the stage of BRCA1 E3 ligase activity after DSB and are awaiting the script of BRCA1 substrate and their roles in the pathway.

## Competing interests

Followings are the patents that may relate to the manuscript.

1) [Tomohiko Ohta]: [Carcinostatic method using BRCA1-BARD1 pathway]. [08/11/2005, WO-2005073379]

2) [Wenwen Wu, and Tomohiko Ohta]: [A method for the ubiquitination of common subunits of RNA polymerases]. [04/26/2007, WO-2007046538]

## Authors' contributions

WW carried out the molecular biological and the biochemical studies that support the conclusions or the proposals. AK and TT has made substantial contributions to analysis and interpretation of the data. TO designed and conducted the studies, and completed the manuscript. All authors read and approved the final manuscript.

## References

[B1] Perou CM, Sorlie T, Eisen MB, van de Rijn M, Jeffrey SS, Rees CA, Pollack JR, Ross DT, Johnsen H, Akslen LA, Fluge O, Pergamenschikov A, Williams C, Zhu SX, Lonning PE, Borresen-Dale AL, Brown PO, Botstein D (2000). Molecular portraits of human breast tumours. Nature.

[B2] Sorlie T, Tibshirani R, Parker J, Hastie T, Marron JS, Nobel A, Deng S, Johnsen H, Pesich R, Geisler S, Demeter J, Perou CM, Lonning PE, Brown PO, Borresen-Dale AL, Botstein D (2003). Repeated observation of breast tumor subtypes in independent gene expression data sets. Proc Natl Acad Sci U S A.

[B3] Nielsen TO, Hsu FD, Jensen K, Cheang M, Karaca G, Hu Z, Hernandez-Boussard T, Livasy C, Cowan D, Dressler L, Akslen LA, Ragaz J, Gown AM, Gilks CB, van de Rijn M, Perou CM (2004). Immunohistochemical and clinical characterization of the basal-like subtype of invasive breast carcinoma. Clin Cancer Res.

[B4] Foulkes WD, Stefansson IM, Chappuis PO, Begin LR, Goffin JR, Wong N, Trudel M, Akslen LA (2003). Germline BRCA1 mutations and a basal epithelial phenotype in breast cancer. J Natl Cancer Inst.

[B5] Lakhani SR, Reis-Filho JS, Fulford L, Penault-Llorca F, van der Vijver M, Parry S, Bishop T, Benitez J, Rivas C, Bignon YJ, Chang-Claude J, Hamann U, Cornelisse CJ, Devilee P, Beckmann MW, Nestle-Kramling C, Daly PA, Haites N, Varley J, Lalloo F, Evans G, Maugard C, Meijers-Heijboer H, Klijn JG, Olah E, Gusterson BA, Pilotti S, Radice P, Scherneck S, Sobol H, Jacquemier J, Wagner T, Peto J, Stratton MR, McGuffog L, Easton DF (2005). Prediction of BRCA1 status in patients with breast cancer using estrogen receptor and basal phenotype. Clin Cancer Res.

[B6] Honrado E, Osorio A, Palacios J, Benitez J (2006). Pathology and gene expression of hereditary breast tumors associated with BRCA1, BRCA2 and CHEK2 gene mutations. Oncogene.

[B7] Turner N, Tutt A, Ashworth A (2004). Hallmarks of 'BRCAness' in sporadic cancers. Nat Rev Cancer.

[B8] Staff S, Isola J, Tanner M (2003). Haplo-insufficiency of BRCA1 in sporadic breast cancer. Cancer Res.

[B9] Turner NC, Reis-Filho JS, Russell AM, Springall RJ, Ryder K, Steele D, Savage K, Gillett CE, Schmitt FC, Ashworth A, Tutt AN (2007). BRCA1 dysfunction in sporadic basal-like breast cancer. Oncogene.

[B10] Richardson AL, Wang ZC, De Nicolo A, Lu X, Brown M, Miron A, Liao X, Iglehart JD, Livingston DM, Ganesan S (2006). X chromosomal abnormalities in basal-like human breast cancer. Cancer Cell.

[B11] Liu X, Holstege H, van der Gulden H, Treur-Mulder M, Zevenhoven J, Velds A, Kerkhoven RM, van Vliet MH, Wessels LF, Peterse JL, Berns A, Jonkers J (2007). Somatic loss of BRCA1 and p53 in mice induces mammary tumors with features of human BRCA1-mutated basal-like breast cancer. Proc Natl Acad Sci U S A.

[B12] McCarthy A, Savage K, Gabriel A, Naceur C, Reis-Filho JS, Ashworth A (2007). A mouse model of basal-like breast carcinoma with metaplastic elements. J Pathol.

[B13] Thompson LH, Schild D (2001). Homologous recombinational repair of DNA ensures mammalian chromosome stability. Mutat Res.

[B14] O'Driscoll M, Jeggo PA (2006). The role of double-strand break repair - insights from human genetics. Nat Rev Genet.

[B15] Hashizume R, Fukuda M, Maeda I, Nishikawa H, Oyake D, Yabuki Y, Ogata H, Ohta T (2001). The RING heterodimer BRCA1-BARD1 is a ubiquitin ligase inactivated by a breast cancer-derived mutation. J Biol Chem.

[B16] Ruffner H, Joazeiro CA, Hemmati D, Hunter T, Verma IM (2001). Cancer-predisposing mutations within the RING domain of BRCA1: loss of ubiquitin protein ligase activity and protection from radiation hypersensitivity. Proc Natl Acad Sci U S A.

[B17] Baer R, Ludwig T (2002). The BRCA1/BARD1 heterodimer, a tumor suppressor complex with ubiquitin E3 ligase activity. Curr Opin Genet Dev.

[B18] Wang B, Matsuoka S, Ballif BA, Zhang D, Smogorzewska A, Gygi SP, Elledge SJ (2007). Abraxas and RAP80 form a BRCA1 protein complex required for the DNA damage response. Science.

[B19] Sobhian B, Shao G, Lilli DR, Culhane AC, Moreau LA, Xia B, Livingston DM, Greenberg RA (2007). RAP80 targets BRCA1 to specific ubiquitin structures at DNA damage sites. Science.

[B20] Kim H, Chen J, Yu X (2007). Ubiquitin-binding protein RAP80 mediates BRCA1-dependent DNA damage response. Science.

[B21] Yan J, Kim YS, Yang XP, Li LP, Liao G, Xia F, Jetten AM (2007). The ubiquitin-interacting motif containing protein RAP80 interacts with BRCA1 and functions in DNA damage repair response. Cancer Res.

[B22] Greenberg RA, Sobhian B, Pathania S, Cantor SB, Nakatani Y, Livingston DM (2006). Multifactorial contributions to an acute DNA damage response by BRCA1/BARD1-containing complexes. Genes Dev.

[B23] Chen J, Silver DP, Walpita D, Cantor SB, Gazdar AF, Tomlinson G, Couch FJ, Weber BL, Ashley T, Livingston DM, Scully R (1998). Stable interaction between the products of the BRCA1 and BRCA2 tumor suppressor genes in mitotic and meiotic cells. Mol Cell.

[B24] Scully R, Anderson SF, Chao DM, Wei W, Ye L, Young RA, Livingston DM, Parvin JD (1997). BRCA1 is a component of the RNA polymerase II holoenzyme. Proc Natl Acad Sci U S A.

[B25] Zhong Q, Chen CF, Li S, Chen Y, Wang CC, Xiao J, Chen PL, Sharp ZD, Lee WH (1999). Association of BRCA1 with the hRad50-hMre11-p95 complex and the DNA damage response. Science.

[B26] Scully R, Chen J, Plug A, Xiao Y, Weaver D, Feunteun J, Ashley T, Livingston DM (1997). Association of BRCA1 with Rad51 in mitotic and meiotic cells. Cell.

[B27] Cantor SB, Bell DW, Ganesan S, Kass EM, Drapkin R, Grossman S, Wahrer DC, Sgroi DC, Lane WS, Haber DA, Livingston DM (2001). BACH1, a novel helicase-like protein, interacts directly with BRCA1 and contributes to its DNA repair function. Cell.

[B28] Bochar DA, Wang L, Beniya H, Kinev A, Xue Y, Lane WS, Wang W, Kashanchi F, Shiekhattar R (2000). BRCA1 is associated with a human SWI/SNF-related complex: linking chromatin remodeling to breast cancer. Cell.

[B29] Hsu LC, White RL (1998). BRCA1 is associated with the centrosome during mitosis. Proc Natl Acad Sci U S A.

[B30] Somasundaram K, Zhang H, Zeng YX, Houvras Y, Peng Y, Zhang H, Wu GS, Licht JD, Weber BL, El-Deiry WS (1997). Arrest of the cell cycle by the tumour-suppressor BRCA1 requires the CDK-inhibitor p21WAF1/CiP1. Nature.

[B31] Ouchi T, Monteiro AN, August A, Aaronson SA, Hanafusa H (1998). BRCA1 regulates p53-dependent gene expression. Proc Natl Acad Sci U S A.

[B32] Zhang H, Somasundaram K, Peng Y, Tian H, Zhang H, Bi D, Weber BL, El-Deiry WS (1998). BRCA1 physically associates with p53 and stimulates its transcriptional activity. Oncogene.

[B33] Krum SA, Miranda GA, Lin C, Lane TF (2003). BRCA1 associates with processive RNA polymerase II. J Biol Chem.

[B34] Lane TF (2004). BRCA1 and transcription. Cancer Biol Ther.

[B35] Scully R, Chen J, Ochs RL, Keegan K, Hoekstra M, Feunteun J, Livingston DM (1997). Dynamic changes of BRCA1 subnuclear location and phosphorylation state are initiated by DNA damage. Cell.

[B36] Chen JJ, Silver D, Cantor S, Livingston DM, Scully R (1999). BRCA1, BRCA2, and Rad51 operate in a common DNA damage response pathway. Cancer Res.

[B37] Mizuta R, LaSalle JM, Cheng HL, Shinohara A, Ogawa H, Copeland N, Jenkins NA, Lalande M, Alt FW (1997). RAB22 and RAB163/mouse BRCA2: proteins that specifically interact with the RAD51 protein. Proc Natl Acad Sci U S A.

[B38] Davies AA, Masson JY, McIlwraith MJ, Stasiak AZ, Stasiak A, Venkitaraman AR, West SC (2001). Role of BRCA2 in control of the RAD51 recombination and DNA repair protein. Mol Cell.

[B39] Pellegrini L, Yu DS, Lo T, Anand S, Lee M, Blundell TL, Venkitaraman AR (2002). Insights into DNA recombination from the structure of a RAD51-BRCA2 complex. Nature.

[B40] Wang Y, Cortez D, Yazdi P, Neff N, Elledge SJ, Qin J (2000). BASC, a super complex of BRCA1-associated proteins involved in the recognition and repair of aberrant DNA structures. Genes Dev.

[B41] Manke IA, Lowery DM, Nguyen A, Yaffe MB (2003). BRCT repeats as phosphopeptide-binding modules involved in protein targeting. Science.

[B42] Yu X, Chini CC, He M, Mer G, Chen J (2003). The BRCT domain is a phospho-protein binding domain. Science.

[B43] Venere M, Snyder A, Zgheib O, Halazonetis TD (2007). Phosphorylation of ATR-interacting protein on Ser239 mediates an interaction with breast-ovarian cancer susceptibility 1 and checkpoint function. Cancer Res.

[B44] Brzovic PS, Keeffe JR, Nishikawa H, Miyamoto K, Fox D, Fukuda M, Ohta T, Klevit R (2003). Binding and recognition in the assembly of an active BRCA1/BARD1 ubiquitin-ligase complex. Proc Natl Acad Sci U S A.

[B45] Hershko A, Ciechanover A (1998). The ubiquitin system. Annu Rev Biochem.

[B46] Pickart CM, Eddins MJ (2004). Ubiquitin: structures, functions, mechanisms. Biochim Biophys Acta.

[B47] Miki Y, Swensen J, Shattuck-Eidens D, Futreal PA, Harshman K, Tavtigian S, Liu Q, Cochran C, Bennett LM, Ding W (1994). A strong candidate for the breast and ovarian cancer susceptibility gene BRCA1. Science.

[B48] Wu LC, Wang ZW, Tsan JT, Spillman MA, Phung A, Xu XL, Yang MC, Hwang LY, Bowcock AM, Baer R (1996). Identification of a RING protein that can interact in vivo with the BRCA1 gene product. Nat Genet.

[B49] Yu X, Fu S, Lai M, Baer R, Chen J (2006). BRCA1 ubiquitinates its phosphorylation-dependent binding partner CtIP. Genes Dev.

[B50] Skowyra D, Craig KL, Tyers M, Elledge SJ, Harper JW (1997). F-box proteins are receptors that recruit phosphorylated substrates to the SCF ubiquitin-ligase complex. Cell.

[B51] Ohta T, Michel JJ, Schottelius AJ, Xiong Y (1999). ROC1, a homolog of APC11, represents a family of cullin partners with an associated ubiquitin ligase activity. Mol Cell.

[B52] Kamura T, Koepp DM, Conrad MN, Skowyra D, Moreland RJ, Iliopoulos O, Lane WS, Kaelin WG, Elledge SJ, Conaway RC, Harper JW, Conaway JW (1999). Rbx1, a component of the VHL tumor suppressor complex and SCF ubiquitin ligase. Science.

[B53] Zheng N, Schulman BA, Song L, Miller JJ, Jeffrey PD, Wang P, Chu C, Koepp DM, Elledge SJ, Pagano M, Conaway RC, Conaway JW, Harper JW, Pavletich NP (2002). Structure of the Cul1-Rbx1-Skp1-F boxSkp2 SCF ubiquitin ligase complex. Nature.

[B54] Chen A, Kleiman FE, Manley JL, Ouchi T, Pan ZQ (2002). Autoubiquitination of the BRCA1*BARD1 RING ubiquitin ligase. J Biol Chem.

[B55] Kleiman FE, Wu-Baer F, Fonseca D, Kaneko S, Baer R, Manley JL (2005). BRCA1/BARD1 inhibition of mRNA 3' processing involves targeted degradation of RNA polymerase II. Genes Dev.

[B56] Starita LM, Horwitz AA, Keogh MC, Ishioka C, Parvin JD, Chiba N (2005). BRCA1/BARD1 ubiquitinate phosphorylated RNA polymerase II. J Biol Chem.

[B57] Sato K, Hayami R, Wu W, Nishikawa T, Nishikawa H, Okuda Y, Ogata H, Fukuda M, Ohta T (2004). Nucleophosmin/B23 is a candidate substrate for the BRCA1-BARD1 ubiquitin ligase. J Biol Chem.

[B58] Wu W, Nishikawa H, Hayami R, Sato K, Honda A, Aratani S, Nakajima T, Fukuda M, Ohta T (2007). BRCA1 ubiquitinates RPB8 in response to DNA damage. Cancer Res.

[B59] Eakin CM, Maccoss MJ, Finney GL, Klevit RE (2007). Estrogen receptor alpha is a putative substrate for the BRCA1 ubiquitin ligase. Proc Natl Acad Sci U S A.

[B60] Starita LM, Machida Y, Sankaran S, Elias JE, Griffin K, Schlegel BP, Gygi SP, Parvin JD (2004). BRCA1-dependent ubiquitination of gamma-tubulin regulates centrosome number. Mol Cell Biol.

[B61] Poole AJ, Li Y, Kim Y, Lin SC, Lee WH, Lee EY (2006). Prevention of Brca1-mediated mammary tumorigenesis in mice by a progesterone antagonist. Science.

[B62] Horwitz AA, Affar el B, Heine GF, Shi Y, Parvin JD (2007). A mechanism for transcriptional repression dependent on the BRCA1 E3 ubiquitin ligase. Proc Natl Acad Sci U S A.

[B63] Pickart CM (2004). Back to the future with ubiquitin. Cell.

[B64] Mukhopadhyay D, Riezman H (2007). Proteasome-independent functions of ubiquitin in endocytosis and signaling. Science.

[B65] Ohta T, Fukuda M (2004). Ubiquitin and breast cancer. Oncogene.

[B66] Chau V, Tobias JW, Bachmair A, Marriott D, Ecker DJ, Gonda DK, Varshavsky A (1989). A multiubiquitin chain is confined to specific lysine in a targeted short-lived protein. Science.

[B67] Nishikawa H, Ooka S, Sato K, Arima K, Okamoto J, Klevit RE, Fukuda M, Ohta T (2004). Mass spectrometric and mutational analyses reveal Lys-6-linked polyubiquitin chains catalyzed by BRCA1-BARD1 ubiquitin ligase. J Biol Chem.

[B68] Wu-Baer F, Lagrazon K, Yuan W, Baer R (2003). The BRCA1/BARD1 heterodimer assembles polyubiquitin chains through an unconventional linkage involving lysine residue K6 of ubiquitin. J Biol Chem.

[B69] Morris JR, Solomon E (2004). BRCA1 : BARD1 induces the formation of conjugated ubiquitin structures, dependent on K6 of ubiquitin, in cells during DNA replication and repair. Hum Mol Genet.

[B70] Mallery DL, Vandenberg CJ, Hiom K (2002). Activation of the E3 ligase function of the BRCA1/BARD1 complex by polyubiquitin chains. Embo J.

[B71] Christensen DE, Brzovic PS, Klevit RE (2007). E2-BRCA1 RING interactions dictate synthesis of mono- or specific polyubiquitin chain linkages. Nat Struct Mol Biol.

[B72] Zhao GY, Sonoda E, Barber LJ, Oka H, Murakawa Y, Yamada K, Ikura T, Wang X, Kobayashi M, Yamamoto K, Boulton SJ, Takeda S (2007). A critical role for the ubiquitin-conjugating enzyme Ubc13 in initiating homologous recombination. Mol Cell.

[B73] Yan J, Kim YS, Yang XP, Albers M, Koegl M, Jetten AM (2007). Ubiquitin-interaction motifs of RAP80 are critical in its regulation of estrogen receptor alpha. Nucleic Acids Res.

[B74] Mailand N, Bekker-Jensen S, Faustrup H, Melander F, Bartek J, Lukas C, Lukas J (2007). RNF8 Ubiquitylates Histones at DNA Double-Strand Breaks and Promotes Assembly of Repair Proteins. Cell.

[B75] Huen MS, Grant R, Manke I, Minn K, Yu X, Yaffe MB, Chen J (2007). RNF8 Transduces the DNA-Damage Signal via Histone Ubiquitylation and Checkpoint Protein Assembly. Cell.

[B76] Kolas NK, Chapman JR, Nakada S, Ylanko J, Chahwan R, Sweeney FD, Panier S, Mendez M, Wildenhain J, Thomson TM, Pelletier L, Jackson SP, Durocher D (2007). Orchestration of the DNA-damage response by the RNF8 ubiquitin ligase. Science.

[B77] Wang B, Elledge SJ (2007). Ubc13/Rnf8 ubiquitin ligases control foci formation of the Rap80/Abraxas/Brca1/Brcc36 complex in response to DNA damage. Proc Natl Acad Sci U S A.

[B78] Plans V, Scheper J, Soler M, Loukili N, Okano Y, Thomson TM (2006). The RING finger protein RNF8 recruits UBC13 for lysine 63-based self polyubiquitylation. J Cell Biochem.

[B79] Ulrich HD, Jentsch S (2000). Two RING finger proteins mediate cooperation between ubiquitin-conjugating enzymes in DNA repair. Embo J.

[B80] Hoege C, Pfander B, Moldovan GL, Pyrowolakis G, Jentsch S (2002). RAD6-dependent DNA repair is linked to modification of PCNA by ubiquitin and SUMO. Nature.

[B81] Stelter P, Ulrich HD (2003). Control of spontaneous and damage-induced mutagenesis by SUMO and ubiquitin conjugation. Nature.

[B82] Unk I, Hajdu I, Fatyol K, Szakal B, Blastyak A, Bermudez V, Hurwitz J, Prakash L, Prakash S, Haracska L (2006). Human SHPRH is a ubiquitin ligase for Mms2-Ubc13-dependent polyubiquitylation of proliferating cell nuclear antigen. Proc Natl Acad Sci U S A.

[B83] Bothos J, Summers MK, Venere M, Scolnick DM, Halazonetis TD (2003). The Chfr mitotic checkpoint protein functions with Ubc13-Mms2 to form Lys63-linked polyubiquitin chains. Oncogene.

[B84] Wooff J, Pastushok L, Hanna M, Fu Y, Xiao W (2004). The TRAF6 RING finger domain mediates physical interaction with Ubc13. FEBS Lett.

